# The Effect of Dexamethasone, Adrenergic and Cholinergic Receptor Agonists on Phospholipid Metabolism in Human Osteoarthritic Synoviocytes

**DOI:** 10.3390/ijms20020342

**Published:** 2019-01-15

**Authors:** Katarzyna D. Sluzalska, Gerhard Liebisch, Bernd Ishaque, Gerd Schmitz, Markus Rickert, Juergen Steinmeyer

**Affiliations:** 1 Laboratory for Experimental Orthopaedics, Department of Orthopaedics, Justus Liebig University Giessen, 35392 Giessen, Germany; K.Sluzalska@gmail.com (K.D.S.); Bernd.Ishaque@ortho.med.uni-giessen.de (B.I.); Markus.Rickert@ortho.med.uni-giessen.de (M.R.); 2 Department of Clinical Chemistry and Laboratory Medicine, University Hospital Regensburg, 93053 Regensburg, Germany; Gerhard.Liebisch@klinik.uni-regensburg.de (G.L.); Gerd.Schmitz@klinik.uni-regensburg.de (G.S.)

**Keywords:** dexamethasone, fibroblast-like synoviocytes, synoviocytes, osteoarthritis, phospholipids, sphingolipids, epinephrine, terbutaline, carbachol, pilocarpine

## Abstract

Phospholipids (PLs) possess the unique ability to contribute to synovial joint lubrication. The aim of our study was to determine for the first time the effect of dexamethasone and some adrenergic and cholinergic agonists on the biosynthesis and release of PLs from human fibroblast-like synoviocytes (FLS). Osteoarthritic human knee FLS were treated with dexamethasone, terbutaline, epinephrine, carbachol, and pilocarpine, or the glucocorticoid receptor antagonist RU 486. Simultaneously PL biosynthesis was determined through the incorporation of stable isotope-labeled precursors into PLs. Radioactive isotope-labeled precursors were used to radiolabel PLs for the subsequent quantification of their release into nutrient media. Lipids were extracted and quantified using electrospray ionization tandem mass spectrometry or liquid scintillation counting. Dexamethasone significantly decreased the biosynthesis of phosphatidylcholine, phosphatidylethanolamine (PE), PE-based plasmalogen, and sphingomyelin. The addition of RU 486 abolished these effects. A release of PLs from FLS into nutrient media was not recognized by any of the tested agents. None of the adrenergic or cholinergic receptor agonists modulated the PL biosynthesis. We demonstrate for the first time an inhibitory effect of dexamethasone on the PL biosynthesis of FLS from human knees. Moreover, our study indicates that the PL metabolism of synovial joints and lungs are differently regulated.

## 1. Introduction

Phospholipids (PLs) are important molecules that participate in many biological processes. Due to their structure, they have the ability to reduce surface tension to very low levels [1,2]. This feature makes them perfect candidates as lung surfactants as well as synovial joint lubricants. PLs are mostly synthesized at the cytosolic side of the endoplasmic reticulum, stored in lamellar bodies, and released from the cells [3]. The alveolar type II cells of the pulmonary system produce and secrete PLs, proteins, and neutral lipids, as well as carbohydrates functioning as surfactants at liquid-air interfaces [4,5]. Phosphatidylcholine (PC) has been found to be the major PL class, constituting 80% of the surfactant lipids [6], from which the most important is saturated dipalmitoyl-phosphatidylcholine (DPPC, PC 32:0) [7]. Several studies reported that fibroblast-like synoviocytes (FLS) are able to synthesize and release synovial joint lubricating molecules such as PLs, hyaluronan, and lubricin [2,8,9,10,11]. We previously reported that PC is the predominant PL class, accounting for 67% of all PLs within human synovial fluid. Also, unsaturated PC species, such as PC 34:1, PC 34:2, PC 36:1, and PC 36:2, are the most common representatives of this class [12]. Our group was also able to demonstrate an association between alterations in synovial lubricant composition and the disease state of the joint [11]. 

It is well known that corticosteroids stimulate the synthesis and secretion of pulmonary surfactants [13,14]. Dexamethasone is often used to enhance lung maturation and promote the production and secretion of lubricants [15]. Also, adrenergic and cholinergic agonists have been found to stimulate pulmonary surfactant synthesis and release [16,17,18,19]. In the lungs, adrenergic agonists act directly on alveolar type II cells, which are responsible for synthesizing and secreting surfactants, as well as interstitial fibroblasts. Cholinergic agonists act indirectly on lung surfactant synthesis and release by stimulating the release of epinephrine by adrenal glands or by contracting smooth muscle cells which in turn stimulates surfactant secretion [14,20]. 

Moreover, sympathetic nerve fibers have been identified in synovium, meniscus, and subchondral bone [21]. Several studies have shown that sympathetic neurotransmitters such as norepinephrine affect chondrogenic differentiation and might accelerate cartilage hypertrophy [21,22]. Also, the sympathetic nervous system affects bone homeostasis by accelerating bone loss, bone remodeling, and osteogenic differentiation [23]. In synovial cells, substance P mediates joint inflammation by promoting the secretion of prostaglandin E2, several matrix metalloproteinases, interleukin-1β (IL-1β), and tumor necrosis factor α (TNFα) [24]. Furthermore, a non-neuronal cholinergic system has been reported to be present in synovial tissue and cartilage of articular joints of patients with rheumatoid arthritis (RA) and osteoarthritis (OA) [25,26]. In M3 muscarinic acetylcholine receptor-deficient mice, the inflammatory response to collagen antibody-induced arthritis tended to be higher than in wild-type littermates [27]. In another study, nicotine prevented cartilage damage and exerted an anti-inflammatory effect in a rat model of OA [28]. Taken together, recent research findings provide evidence for the importance of sympathetic and parasympathetic structures and neurotransmitters in inflammatory joint diseases.

Dexamethasone is a potent synthetic corticosteroid which is often used in the treatment of lung and rheumatic disorders, inflammation, allergies, and asthma. Intra-articular injections of dexamethasone are used for OA and RA treatment [29,30]. Several studies have shown that dexamethasone inhibits the induction of matrix metalloproteinases, prostaglandins, inflammatory cytokines, and oxygen-derived radicals [31,32,33,34,35,36]. Dexamethasone decreased the synthesis and secretion of inflammatory factors from TNFα-treated FLS [37,38]. It also inhibited IL-1β-induced phospholipase A2, prostaglandin E2, and cyclooxygenase (COX) activity in human FLS [39]. This corticoid interferes with NF-κB and apoptosis pathways and has also been found to inhibit synovial inflammation [40,41]. Interestingly, intra-articular administration of methylprednisolone acetate, another corticosteroid, was reported to induce an elevated release of PLs into equine synovial fluids and in so doing was suggested to improve joint mobility [42]. We previously reported that PLs are part of the lubricating system and that elevated levels of PLs were found in the knee synovial fluid of patients with OA and RA [11]. However, currently, no detailed data are available regarding the impact of dexamethasone on PL metabolism in FLS obtained from human osteoarthritic joints. 

Given this lack of information and the reported association between dexamethasone and pulmonary surfactant production, we sought to evaluate the effect of this corticosteroid on PL metabolism in human FLS from OA knees. The aim of our study was to investigate for the first time the individual effects which dexamethasone, as well as agonists of adrenergic and muscarinic receptors, have on PL classes and species synthesized and released by human FLS. Based on our analysis, we provide further insights into the regulatory mechanisms controlling PL metabolism in articular joints. 

## 2. Results

### 2.1. The Effect of Dexamethasone on the Biosynthesis of PL Classes

**Figure 1:**
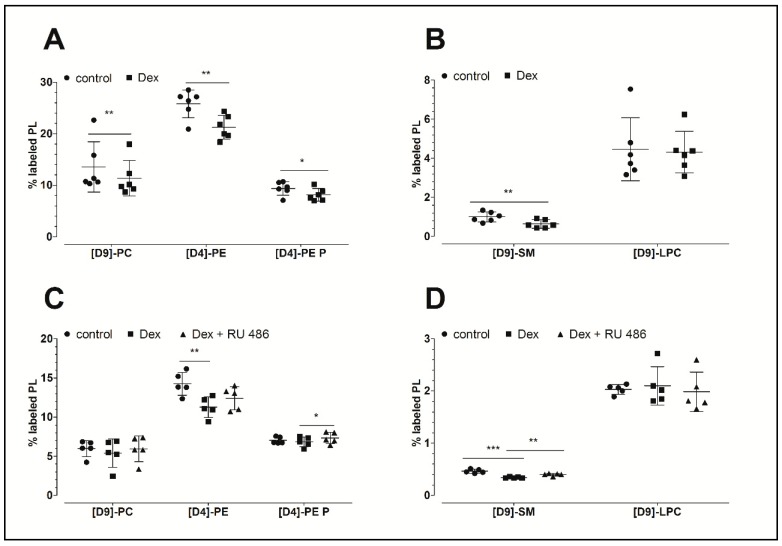
The effect of dexamethasone and RU 486 on the biosynthesis of PLs by human FLS. (**A**,**B**) The effect of dexamethasone on the biosynthesis of PL classes. The percentages of labeled PL classes from the total corresponding labeled and unlabeled PL classes are presented. FLS were treated with 10 µM dexamethasone (Dex) for 16 h. Data are presented as means ± SDs (*n* = 6). (**C**,**D**) The effect of dexamethasone on the biosynthesis of PL classes as modulated by the glucocorticoid receptor antagonist RU 486. The percentages of labeled PL classes from the total corresponding labeled and unlabeled PL classes are presented. FLS were first pretreated for 30 min with 1 µM RU 486, and then treated with 10 µM dexamethasone (Dex) for 16 h in the presence of stable isotope-labeled lipid precursors. Data are presented as means ± SDs (*n* = 5). * *p* ≤ 0.05, ** *p* ≤ 0.01, *** *p* ≤ 0.001. PC = phosphatidylcholine; PE = phosphatidylethanolamine; PE P = phosphatidylethanolamine-based plasmalogens; SM = sphingomyelin; LPC = lysophosphatidylcholine.

Figure 1A,B demonstrates that dexamethasone inhibits PL biosynthesis. This glucocorticoid significantly decreased the biosynthesis of phosphatidylcholine to 84% (PC: 11.4 ± 3.5%, *p* = 0.004; 10.7 ± 3.1 nmol/mg protein), the biosynthesis of phosphatidylethanolamine to 82% (PE: 21.3 ± 2.3%, *p* = 0.005; 6.6 ± 2.4 nmol/mg protein), the biosynthesis of PE-based plasmalogen to 87% (PE P: 8.1 ± 1.2%, *p* = 0.021; 2.1 ± 0.4 nmol/mg protein), and the biosynthesis of sphingomyelin to 64% (SM: 0.64 ± 0.22%, *p* = 0.003; 162 ± 56 pmol/mg protein) compared to the untreated controls (PC: 13.6 ± 4.9%, 12.8 ± 4.7 nmol/mg protein; PE: 25.8 ± 2.7%, 8.2 ± 1.6 nmol/mg protein; PE P: 9.4 ± 1.3%, 2.2 ± 0.2 nmol/mg protein; and SM: 1.0 ± 0.3%, 245 ± 74 pmol/mg protein, respectively). Since SM derives from PC, the ratios of newly synthesized SM to its precursor, namely the newly synthesized PC, were calculated. The analysis revealed decreased ratios for dexamethasone treatments (0.06 ± 0.02) when compared to untreated controls (0.08 ± 0.02) which indicates a specific inhibitory effect of the dexamethasone. The biosynthesis of lysophosphatidylcholine (LPC) remained unchanged upon dexamethasone treatment (dexamethasone: 4.3 ± 1.1%, 80 ± 20 pmol/mg protein; control: 4.5 ± 1.6%: 65 ± 23 pmol/mg protein). Table 1 summarizes the concentrations of newly synthesized PL classes. 

### 2.2. The Effect of the Glucocorticoid Receptor Antagonist RU 486

In a separate experiment, our analysis focused on the possible mechanism of action of dexamethasone. As shown in Figure 1C,D, dexamethasone again decreased the biosynthesis of PE to 79% (11.3 ± 1.3%, *p* = 0.003; 2.06 ± 0.29 nmol/mg protein) and that of SM to 74% (0.34 ± 0.01%, *p* < 0.001, 87 ± 22 pmol/mg protein) when compared to untreated controls (PE: 14.3 ± 1.5%, 2.90 ± 0.55 nmol/mg protein; SM: 0.46 ± 0.04%, 115 ± 28 pmol/mg protein, respectively). In addition, a slight but non-significantly decreased synthesis of PC and PE-based plasmalogen was observed. Once again, the biosynthesis of LPC remained unchanged in response to dexamethasone treatment (dexamethasone: 2.1 ± 0.37%, 27 ± 7 pmol/mg protein; control: 2.0 ± 0.09%, 27 ± 8 pmol/mg protein). Furthermore, the blockade of the glucocorticoid receptor with RU 486 abolished the dexamethasone effect on the biosynthesis of SM (*p* = 0.007). Since SM derives from PC, the ratios of the newly synthesized SM to its newly synthesized precursor PC were calculated. Our analysis revealed no altered ratios upon treatment, suggesting no specific effect of RU 486 on the biosynthesis of SM. The levels of newly synthesized PL classes are presented in Table 1.

### 2.3. A Detailed Analysis of the Dexamethasone Effect on PL Species

**Figure 2:**
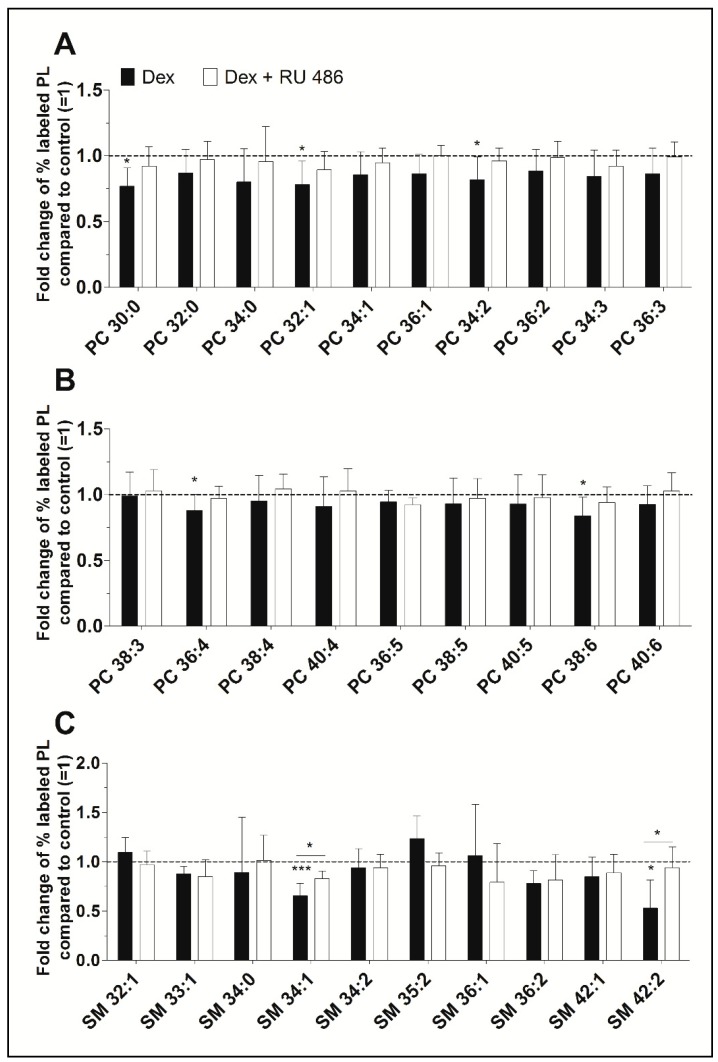
(**A**,**B**) The effect of dexamethasone on the synthesis of individual PC and (**C**) SM species as modulated by the glucocorticoid receptor antagonist RU 486. PL biosynthesis was monitored with ESI-MS/MS in the presence of 10 µM dexamethasone (Dex, black bars) alone or together with 1 µM RU 486 (white bars) for 16 h (*n* = 5). The percentages of stable isotope-labeled PC species were calculated and then normalized as ratios of the corresponding untreated controls. As such, data are presented as means ± SDs of the x-fold change of % labeled PL species compared to untreated controls (=1). * *p* ≤ 0.05, *** *p* ≤ 0.001. PC = phosphatidylcholine; SM = sphingomyelin.

Our ESI-MS/MS analysis allowed us to determine whether dexamethasone affects the biosynthesis of specific PC species. Their concentrations varied between 39 ± 5 pmol/mg protein (PC 34:3) and 861 ± 90 pmol/mg protein (PC 34:1) for untreated controls and between 28 ± 8 pmol/mg protein (PC 34:3) and 726 ± 206 pmol/mg protein (PC 34:1) after treatment with dexamethasone (Table S1). Dexamethasone significantly decreased the synthesis of five PC species, namely PC 30:0, to 77% (*p* = 0.014), PC 32:1 to 78% (*p* = 0.037), PC 34:2 to 82% (*p* = 0.049), PC 36:4 to 88% (*p* = 0.046), and PC 38:6 to 84% (*p* = 0.032). Figure 2A,B shows, that the glucocorticoid receptor with RU 486 appears to antagonize this inhibitory effect. Also, over 86% of newly synthesized PC species were unsaturated irrespective of the treatment. The length of the FA chains of newly synthesized PC species did not differ between treated and untreated FLS: 77.3 ± 4.2% had equal to or less than 36 carbon atoms in untreated FLS, 75.8 ± 5.0% had equal to or less than 36 carbon atoms in FLS treated with dexamethasone, and 76.7 ± 4.8% had equal to or less than 36 carbon atoms after treatment with dexamethasone in the presence of the glucocorticoid receptor antagonist RU 486.

Newly synthesized SM species showed concentrations varying between 3 ± 1 pmol/mg protein (SM 36:2) and 54 ± 8 pmol/mg protein (SM 34:1) for untreated controls and between 2 ± 0 pmol/mg protein (SM 36:2) and 37 ± 11 pmol/mg protein (SM 34:1) where cells were treated with dexamethasone (Table S1). Dexamethasone significantly inhibited the biosynthesis of two SM species, namely SM 34:1 (to 65%, *p* = 0.001) and SM 42:2 (to 53%, *p* = 0.019). The blockade of the glucocorticoid receptor with RU 486 abolished this effect (Figure 2C).

**Figure 3:**
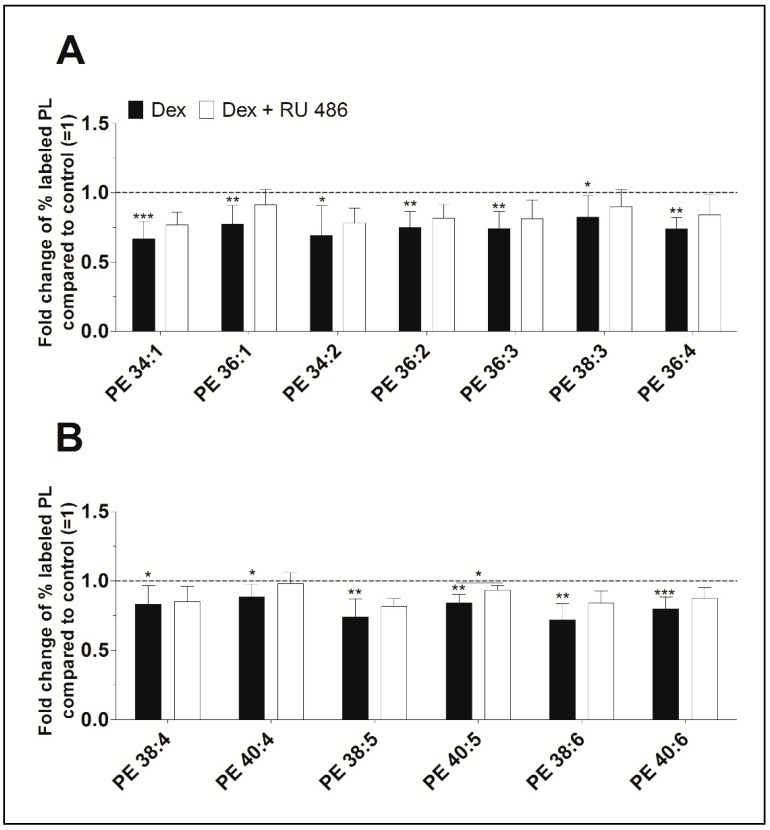
The effect of dexamethasone on the biosynthesis of the PE species (**A**) PE 34:1 to PE 36:4, and (**B**) PE 38:4 to PE 40:6 as modulated by the glucocorticoid receptor antagonist RU 486. PE biosynthesis was monitored by ESI-MS/MS in the presence of dexamethasone (Dex, black bars) alone or together with RU 486 (white bars) for 16 h (*n* = 5). The percentages of stable isotope-labeled PE species were calculated and then normalized as ratios of the corresponding untreated controls. Thus, data are presented as means ± SDs of the x-fold change of % labeled PE species compared to the untreated controls (=1). * *p* ≤ 0.05, ** *p* ≤ 0.01, *** *p* ≤ 0.001. PE = phosphatidylethanolamine.

Furthermore, newly synthesized PE species displayed concentrations varying between 37 ± 16 pmol/mg protein (PE 34:2) and 722 ± 134 pmol/mg protein (PE 38:4) for untreated control, and between 18 ± 8 pmol/mg protein (PE 34:2) and 558 ± 125 pmol/mg protein (PE 38:4) after treatment with dexamethasone (Table S1). As shown in Figure 3, dexamethasone significantly decreased the biosynthesis of all PE species between 88% (PE 40:4, *p* = 0.022) and 67% (PE 34:1, *p* < 0.001) when compared to untreated controls. Blockade of the glucocorticoid receptor with RU 486 slightly abolished the dexamethasone effect on the synthesis of PE. All newly synthesized PE species were unsaturated. Also, the length of the FA chains of newly synthesized PE species did not markedly differ between untreated and treated FLS and were as follows: 84.5 ± 4.5% had equal to or more than 36 carbon atoms in untreated FLS, 86.6 ± 4.0% had equal to or more than 36 carbon atoms in FLS treated with dexamethasone, and 86.4 ± 4.1% had equal to or more than 36 carbon atoms in cells being treated with dexamethasone and RU 486.

Dexamethasone did not affect the biosynthesis of any LPC species (Table S1). Only three LPC species, namely LPC 16:0, LPC 18:0, and LPC 18:1, were detected at low concentrations of about 10 pmol/mg proteins. Also, the biosynthesis of nineteen detected PE-based plasmalogen species remained unaffected by dexamethasone (Table S1). 

### 2.4. The Effect of Adrenergic and Muscarinic Receptor Agonists on PL Biosynthesis 

Our further study was stimulated by recent findings on the role of the neurotransmitters of the autonomic nervous system within articular joints. We focused on receptor agonists of the sympathetic and parasympathetic nervous system to see whether they can affect PL biosynthesis in FLS. Our data reveal that the adrenergic receptor agonists terbutaline and epinephrine, as well as the muscarinic receptor agonists carbachol and pilocarpine, exert no or only weak effects on PL synthesis (Table 1). Only pilocarpine slightly increased the biosynthesis of the PE as a class to 108% when compared to untreated FLS (untreated controls: 22.6 ± 2.9%, 7.6 ± 2.6 nmol/mg protein; pilocarpine treated FLS: 24.5 ± 2.7%, *p* = 0.030, 8.4 ± 3.7 nmol/mg protein). 

Our in-depth analysis investigated whether certain PL species are individually affected by any of the receptor agonists being tested. Interestingly, we found that compared with untreated controls, pilocarpine stimulated the synthesis of PE 38:3 to 108% (*p* = 0.003), PE 38:4 to 112% (*p* = 0.002), and PE 40:4 to 108% (*p* = 0.038), while terbutaline increased the biosynthesis of PE 36:1 to 113% (*p* = 0.026), PE 38:3 to 108% (*p* = 0.025), and PE 38:4 to 110% (*p* = 0.027). Also, carbachol enhanced the synthesis of PE 36:1 and PE 38:3 to 112% (*p* = 0.033) and 114% (*p* < 0.001), respectively, while epinephrine increased only the biosynthesis of one PE, namely PE 38:3 (107%, *p* = 0.020). 

Moreover, terbutaline, epinephrine, and pilocarpine respectively enhanced the biosynthesis of the LPC class to 128% (*p* = 0.019), 138% (*p* = 0.017), and 136% (*p* = 0.010) (Table 1). Remarkably, our in-depth analysis revealed that the synthesis of only one species, namely LPC 18:0, was significantly affected by terbutaline (5.8 ± 1.8%, *p* = 0.003; 30 ± 10 pmol/mg protein), epinephrine (5.9 ± 2.3%, *p* = 0.018; 29 ± 11 pmol/mg protein) and pilocarpine (5.8 ± 2.9, *p* = 0.013; 35 ± 16 pmol/mg protein) when compared to untreated controls (4.1 ± 1.6%; 21 ± 9 pmol/mg protein) (Table S2). The levels of LPC species being detected in nutrient media were low within the range of 17–35 pmol/mg protein. The biosynthesis of individual species of PC, SM, and PE-based plasmalogen remained unchanged upon treatment with receptor agonists.

### 2.5. The In Vitro Model of FLS to Study PL Release

**Figure 4:**
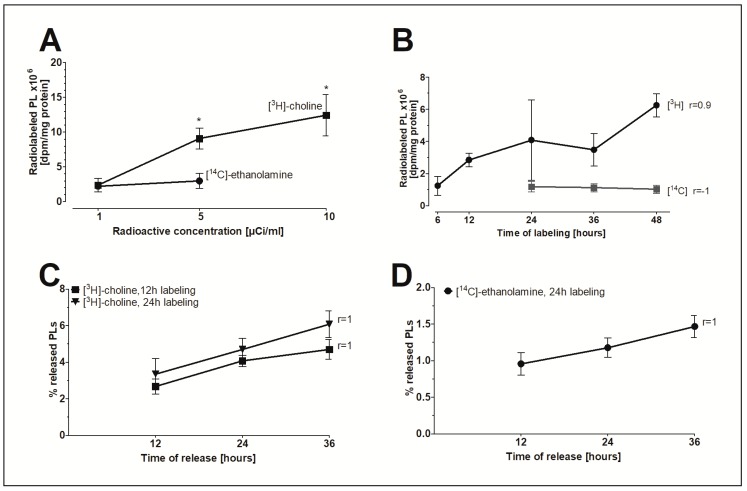
The in vitro model of FLS to study the release of PLs. (**A**) The concentration-dependent effect and (**B**) the time-dependent effect of the incorporation of radioactive precursors into the PLs of FLS. (**C**) The release of [^3^H]-choline-labeled PLs and (**D**) [^14^C]-ethanolamine-labeled PLs from FLS into media as a function of time. FLS were initially labeled with 5 µCi/mL of [^3^H]-choline and 1 µCi/mL of [^14^C]-ethanolamine for 12 and/or 24 h. The release of radiolabeled PLs into media with 2% FBS was then monitored during a 12–36 h period. The dpm-values were normalized to the cellular protein content. Data shown represent the percentages of released radiolabeled PLs from total radiolabeled PLs as found in media and cellular lysates. Data are presented as means ± SDs (*n* = 3). * *p* ≤ 0.05.

To investigate whether FLS are a possible source for extracellular PLs, an in vitro model to study the release of PLs was established. The radioactive isotope-labeled precursors [^3^H]-choline and [^14^C]-ethanolamine were initially incorporated into PLs to study the release of radiolabeled PLs under the influence of various agents. Figure 4A shows that increasing the radioactive concentration of [^3^H]-choline but not [^14^C]-ethanolamine in the nutrient media from 1 to 5 or 10µCi/mL significantly elevated the pool of radioisotope-labeled PLs in FLS. For this reason, specific activities of 5µCi/mL [^3^H]-choline and 1 µCi/mL [^14^C]-ethanolamine were ultimately chosen for the in vitro model. Figure 4B demonstrates that only the amount of [^3^H]-choline-labelled lipids correlates with the time of labeling. The release of [^3^H]-choline-labeled (Figure 4C) and [^14^C]-ethanolamine-labeled PLs (Figure 4D) from human FLS into DMEM containing 2% FBS increased linearly with time (*r* = 1). In conclusion, a 24-h labeling period was chosen to ensure a measurable level of radiolabeled PLs in nutrient media and to minimize the amount of PLs being internalized by the cells. Furthermore, a 24-h period of linear release of radiolabeled PLs was chosen to determine the effect of the agents on PL release. 

### 2.6. The Release of PLs from FLS as Modulated by Agonists

To investigate the effects of various agents on the release of radiolabeled PLs, FLS were treated with dexamethasone, terbutaline, epinephrine, carbachol, and pilocarpine during the 24-h release period. As shown in Table 2, relatively more [^3^H]-choline-labeled PLs were released into the media than [^14^C]-ethanolamine-labeled PLs. However, none of the tested agents were found to modulate the release of PLs. 

## 3. Discussion

Glucocorticoids have been reported to release lubricating surfactants, and particularly PLs, into equine synovial joints [42], and to promote the biosynthesis and release of lung surfactants, including PLs [13,14]. Our present study, therefore, attempted to determine the effect of the corticosteroid dexamethasone on the PL metabolism of human FLS derived from OA knee joints. Our results demonstrate that dexamethasone inhibited the biosynthesis of PLs, but did not influence their release from FLS. Our initial screening experiment revealed that dexamethasone decreased the biosynthesis of PC, PE, PE P, and SM. An additional experiment aimed at blocking the dexamethasone effect using a glucocorticoid receptor antagonist was able to confirm our observation. Our data obtained with knee FLS contradict those obtained with human fetal lungs cultured as an explant, in which dexamethasone significantly stimulated the incorporation of [^3^H]-choline into PC. In the present study using human FLS, dexamethasone actually decreased the incorporation of stable isotope-labeled precursors into PLs. Our data therefore indicate alternative regulatory mechanisms for PL biosynthesis between synovial joints and lungs. 

In our study, dexamethasone was found to be an inhibitor of PE and SM biosynthesis. Apart from their role in maintaining cell membrane integrity, PE species and their metabolites such as diacylglycerol also function as precursors of molecules that modulate pain perception, inflammation, autophagy, and apoptosis [43,44,45,46,47]. Also, SM species and their metabolites including ceramides and sphingosine play roles in cell signaling, apoptosis, and survival [48,49]. As such, lower levels of PE species may, in fact, suppress inflammation by inhibiting the expression of pro-inflammatory cytokines within the synovial joint. We also hypothesize that a reduced level of SM after dexamethasone treatment may counteract the apoptotic process within chondrocytes. Taken together, our data imply a possible beneficial effect of dexamethasone on OA through downregulation of lipid biosynthesis. However, further studies will certainly provide additional insights to confirm these suggested effects of dexamethasone within the joints.

Dexamethasone has also been reported to inhibit synovial inflammation [40,41]. Phosphatidylethanolamine-binding protein-1 has been found to interact with a range of signaling molecules that participate in inflammatory processes [50]. Also, bioactive aldehydes generated from PE have been reported to mediate inflammation [51]. In our previous study, we showed that IL-1β increases the biosynthesis of PE and PE-based plasmalogens. Nine PE and four PE-based plasmalogen species were previously found to be upregulated by IL-1β [52]. Here, we demonstrate that dexamethasone was able to inhibit PE biosynthesis, indicating a possible antagonizing effect of dexamethasone on IL-1ß via the signal transduction pathway of NF-κB in the synovium [41]. 

Interestingly, dexamethasone appears to act only in part through the glucocorticoid receptor. The blockade of the glucocorticoid receptor with RU 486 abolished the dexamethasone effect on the biosynthesis of only one PE and two SM species. Glucocorticoids have been reported to act through two types of nuclear receptors, namely the glucocorticoid receptor NR3C1 and the mineralocorticoid receptor NR3C2 [53]. The response takes place over a course of hours. Glucocorticoids might also rapidly act through membrane-bound receptors [54,55]. Consistently with this, our data suggest that the dexamethasone effect on PL biosynthesis may not only be mediated by the nuclear glucocorticoid receptor. This might be one reason why we were not able to observe a more pronounced inhibitory effect. Further studies are required to elucidate the mechanism of dexamethasone action on PL synthesis in articular joint FLS.

Since intra-articular injections of dexamethasone are commonly used in OA and RA treatments [29,30], we compared PL species that are known to be altered in early OA synovial fluid [12] with those of FLS treated with dexamethasone. We report here that the levels of PLs are regulated in opposite directions. The biosynthesis of nine PE and three PC species which were elevated during early OA was decreased after dexamethasone treatment. Our data together with the reported anti-inflammatory properties [40,41] of dexamethasone suggest a possible therapeutic potential for dexamethasone in OA with the goal of restoring normal PL homeostasis within articular joints. 

Adrenergic and cholinergic receptor agonists have been found to stimulate pulmonary surfactant production and release [14,56,57,58,59]. Moreover, recent findings have provided clear evidence for the presence and (patho)physiological role of the cholinergic and sympathetic nervous systems and their neurotransmitters within human articular joint tissues including synovium, cartilage, and bone during health, OA and RA [21,24,28]. This is why we studied the effect of the adrenergic agonists, terbutaline and epinephrine, as well as the cholinergic agonists, carbachol and pilocarpine, on the biosynthesis of PLs. Our data indicate that these agonists have some effects on the biosynthesis of PE and LPC, but not on PC, SM, and PE-based plasmalogens. Remarkably, pilocarpine, terbutaline, and epinephrine markedly stimulated the biosynthesis of LPC 18:0, which may have a role as an immunomodulatory lipid species. Our data indicate again that different mechanisms are involved in the regulation of PL biosynthesis within synovial joints and lungs.

Since it was unknown how the release of PLs from human FLS is controlled, we then went on to establish an in vitro model. This model was already able to show that newly synthesized PLs containing choline are preferably released into cell culture media, a fact which might explain in part the high amount of PC found within human synovial fluid [12]. Nevertheless, the majority of PLs found in synovial fluid seem to originate from the blood. In the pulmonary surfactant system, dexamethasone, as well as cholinergic and adrenergic agonists, have been reported to stimulate the secretion of surfactants including PLs [13,14]. In our experiment, none of these agents had any effect on PL release. Our data again underline the fact that PLs in synovial joints are differently regulated compared to those which function as pulmonary surfactants. 

Remarkably, Hills et al. reported that methylprednisolone significantly promoted PL secretion into equine synovial fluid [42]. The main lung surfactant lipid species dipalmitoylphosphatidylcholine (DPPC) was used as a standard to evaluate surfactant levels which we later found only in small quantities within human SF as compared to other possible surface-active PC species such as PC 34:1, PC 34:2, and PC 36:2 [12]. However, in our current in vitro study, we were not able to confirm that dexamethasone stimulates the release of PLs from human FLS. This may have been due to species differences or differences between the corticosteroids applied.

In conclusion, we have shown here for the first time that dexamethasone is an inhibitor of PL biosynthesis in FLS from human OA knees. We also established a new model to study the release of PLs which allowed us to show that dexamethasone has no impact on PL release from human FLS. Nevertheless, our data support the therapeutic use of dexamethasone for balancing altered PL compositions during diseases such as OA. Moreover, adrenergic and cholinergic agonists have only minor influences on PE and SM synthesis and do not modulate their release. Our data provide strong evidence that the metabolism of surface-active PLs is differently regulated in synovial joints and lungs. 

## 4. Materials and Methods

### 4.1. Reagents

Unless otherwise indicated, all reagents were purchased from Sigma (Deisenhofen, Germany). Dulbecco’s modified Eagle media (DMEM), Dulbecco’s phosphate buffered saline (PBS) and penicillin/streptomycin were acquired from PAN Biotech (Aidenbach, Germany), HPLC-grade methanol and chloroform were from Merck (Darmstadt, Germany), [methyl,-^3^H]-choline chloride was from PerkinElmer (Waltham, MA, USA), [1,2-^14^C] ethanolamine hydrochloride was from Hartman Analytic (Braunschweig, Germany), and trimethyl-[D9]-choline chloride and [D4]-ethanolamine were from Cambridge Isotope Laboratories (Andover, MA, USA).

### 4.2. Isolation of Fibroblast-Like Synoviocytes (FLS)

FLS were obtained from synovial membranes of OA patients undergoing total knee replacement surgery as described elsewhere [60]. The study was approved on 31 October 2013 by the local ethics committee of the Justus Liebig University Giessen (Az 106/03), and all patients provided informed consent to donate samples for research before the experiments were begun. The effects of dexamethasone, adrenergic and cholinergic agonists on FLS were tested with cells derived from 16 OA patients of both genders (7 male, 9 female), aged 50–85 years (76.1 ± 7.6 years), with BMIs of 20–35 (28.7 ± 2.9 kg/m^2^), Kellgren-Lawrence scores of 3.5 ± 0.52, and CRP values of 6.0 ± 12.7 mg/L. FLS were excluded from patients with (a) other joint diseases such as RA, gout, or trauma, (b) knee joint surgery within the last 6 months prior to study onset, (c) severe diseases such as HIV infection, tumors near to the affected knee joint, severe liver and/or kidney diseases, drug abuse, and (d) intake of immunosuppressive drugs, corticosteroids, or hyaluronan within the last 6 months prior to study onset. 

### 4.3. Cell Culture Procedure 

FLS were cultured in a humidified 10% CO_2_ atmosphere at 37 °C using DMEM medium supplemented with 1.0 g/L glucose, 584 mg/L l-glutamine, 10% fetal bovine serum (FBS), 10 mM HEPES buffer, 10 U/mL penicillin, and 0.1 mg/mL streptomycin. The experiments were performed with cells from passage No. 5. Routine tests for mycoplasma contamination using the PCR Mycoplasma Kit (PromoCell, Heidelberg, Germany) were negative. 

### 4.4. FACS Analysis

The purity of FLS was determined at the end of passage 4–5 with a BD FACSCANTO II flow cytometer (Becton Dickinson, Heidelberg, Germany). After trypsinization, cells were stained with APC anti-human CD90 (clone 5E10) and PE anti-human CD45 (clone 2D1) or APC mouse IgG1 and PE mouse IgG1 antibodies (clone MOPC-21, BioLegend, San Diego, CA, USA). More than 80% of cells used in the experiments were stained positively for the fibroblast-specific antigen CD90 (87.1 ± 18.4%), whereas staining for CD45 was negative. 

### 4.5. The Effect of Dexamethasone, Cholinergic and Adrenergic Agonists on the Biosynthesis of PLs

For the analysis of PL biosynthesis, FLS from passage No. 5 from 6 patients were cultured in 6-well plates at a density of 80,000 FLS per well. Cells were grown until 100% confluency and then starved for 24 h in serine- and choline-depleted, phenol-free DMEM medium (PAN Biotech, Aidenbach, Germany) containing 5% lipoprotein-deficient serum (LPDS, a generous gift from Dr. A. Sigruener), 10 mM HEPES buffer, 10 U/mL penicillin, 0.1 mg/mL streptomycin and 4 mg/L folic acid. The medium was also supplemented with 42 mg/L l-serine to ensure an adequate supply of all amino acids. FLS were treated with 10 µM of dexamethasone (Dex), epinephrine, terbutaline, carbachol, or pilocarpine for 16 h in the presence of 225 µg/mL of [D9]-choline and 25 µg/mL of [D4]-ethanolamine. Untreated FLS obtained from the same joints were used as controls. In a separate set of experiments, we investigated whether blocking the glucocorticoid receptor abolishes the effect of dexamethasone. Here, FLS treated with 10 µM dexamethasone were compared with FLS pretreated for 30 min with 1 µM RU 486 (Selleckchem, Munich, Germany) and then treated with 10 µM dexamethasone. Afterward, media were harvested, cells were washed twice with 1× PBS, and lysed with 0.2% sodium dodecyl sulfate. Wells were washed with distilled water and combined extracts were treated with ultrasound (Sonopuls model UW 2010, Bandelin electronic, Berlin, Germany) for 6 s with 40–50% power. The protein concentrations of cellular lysates were quantified using the Pierce™ BCA Protein Assay Kit (Thermo Fisher, Darmstadt, Germany).

### 4.6. Release Model

PL release was determined in FLS seeded into 6-well plates at a density of 80,000 cells per well. Phenol-free DMEM medium containing 10% FBS, 10 mM HEPES buffer, 10 U/mL penicillin, and 0.1 mg/mL streptomycin was used. FLS were grown until 100% confluency and then labeled for 6–48 h with 1–10 µCi/mL [^3^H]-choline and 1–5 µCi/mL of [^14^C]-ethanolamine. Cells were adapted to low-level FBS in that they were first thoroughly washed to remove unincorporated isotopes, then incubated with DMEM media containing 5% FBS for 24 h, followed by a 24-h culture period in DMEM with 2% FBS. The release of radiolabeled PLs was determined in media collected after 12–36 h from FLS cultured in fresh DMEM containing 2% FBS. Cells were washed twice with 1× PBS, lysed using 0.2% sodium dodecyl sulfate, and treated with ultrasound as described above. Proteins within cellular lysates were quantified using the Pierce™ BCA Protein Assay Kit (Thermo Fisher, Darmstadt, Germany). 

Our preliminary experiments revealed that FLS of the release model maintained a stable metabolism as indicated by the unaltered expression of the reference genes B2M, β-actin, and GAPDH (QuantiTect^®^ Primer Assays, Qiagen, Hilden, Germany), by the constant mitochondrial activity (Cell Titer 96^®^, Promega, Madison, WI, USA), and by the high cell viability (>90%, trypan blue exclusion test, Sigma).

### 4.7. The Effect of Dexamethasone, Cholinergic and Adrenergic Agonists on PL Release

In order to analyze the release of radiolabeled PLs, FLS of the 5th passage from 4–5 patients were used in the release model. During the release of radiolabeled PLs from FLS into nutrient media over 24 h, cells were treated with 10 µM dexamethasone (Dex), epinephrine, terbutaline, carbachol, or pilocarpine. The release was terminated by the sampling of the media and cells were lysed and extracted as described above.

### 4.8. Lipid Extraction

Lipid extraction was performed according to the method of Bligh and Dyer [61] described above, either on stable isotope-labeled cellular lysates in the presence of non-naturally occurring internal lipid standards (Avanti Polar Lipids, Alabaster, AL, USA), or on radioactive isotope-labeled cellular lysates and media samples obtained from the release model without the addition of any standards. 

### 4.9. PL Analysis by Mass Spectrometry

Stable isotope-labeled and unlabeled PL species were quantified using electrospray ionization tandem mass spectrometry (ESI-MS/MS) on a Quattro Ultima™ Triple Quadruple mass spectrometer (Micromass, Wilmslow, UK) as described previously [62]. Briefly, a precursor ion scanning with an m/z of 184 was used for phosphatidylcholine (PC), sphingomyelin (SM), and lysophosphatidylcholine (LPC) detection. [D9]-choline-labeled lipids were analyzed using precursor ion scanning with an m/z of 193. A neutral loss of 141 Da was used for phosphatidylethanolamine (PE) detection. [D4]-ethanolamine-labeled lipids were analyzed by neutral loss of 145 Da. Fragment ions of m/z 364, 390 and 392 were used for detecting phosphatidylethanolamine-based plasmalogens (PE P) PE P-16:0, PE P-18:1, and PE P-18:0. The isotopic overlap of lipid species was corrected and data analysis was performed using self-programmed Excel macros [63]. Lipid species were annotated according to a standard methodology for reporting lipid species identified from mass spectrometry [64]. Glycerophospholipid annotation is based on the assumption of even-numbered carbon chains only. SM species annotation is based on the assumption that a sphingoid base with two hydroxyl groups is present. The quantitative values were normalized to the cellular protein content and are expressed as nmol/mg or pmol/mg protein. Only PL species with concentrations higher than 1% of the corresponding PL class, and more than three times higher than the internal standard blank, were taken into consideration. 

### 4.10. The Release of Radioactive PLs

Radioactive isotope-labeled PLs were quantified using liquid scintillation counting (LSC). The chloroform phases of the lipid extraction performed according to the procedure described by Bligh and Dyer [61] were obtained and mixed with LSC cocktail Emulsifier-Safe™ (Perkin Elmer, Waltham, MA, USA). Samples were thoroughly mixed and subsequently measured using a Multi-Purpose Scintillation Counter LS 6500 (Beckman Coulter, Fullerton, CA, USA) with a [^3^H]- and [^14^C]-dual-label channel setting. The quantitative dpm-values were normalized to the cellular protein content and expressed as dpm/mg cellular protein. The percentages of radiolabeled PLs released from total radiolabeled PLs as found in media and cellular lysates were calculated separately for the [^3^H]- as well as the [^14^C]-labeled PLs.

### 4.11. Statistical Analysis

Each experimental condition was repeated 5–6 times using FLS obtained from 5–6 patients (*n* = 5–6). The data were analyzed as logits of the proportions in a two-factorial linear model. The factor "Patient" accounts for systematic differences between cell cultures obtained from different patients (“paired analysis”). The “Group” factor accounts for differences between treatments. Residual diagnostic plots showed good agreement of the data with the model assumptions. Differences in treatment effects were tested with Tukey’s HSD (Figure 1, Figure 2 and Figure 3, Table 1 and Table 2). Paired *t*-tests were applied to analyze the effect of increasing concentrations of radioactive isotopes on radiolabeled PLs (Figure 4A). Correlations between the time of labeling and PL (Figure 4B) as well as the time of release on PL release (Figure 4C–D) were calculated using Spearman’s rank correlation. The %-values quoted in the text within brackets represent the percentage of the labeled PL class or individual species from the total corresponding PL class or species being determined, both labeled and unlabeled. The analysis was performed in R version 3.3.2 [65]. Graphs were created using Prism 5.2 (GraphPad Software Inc., La Jolla, CA, USA). Data are presented as means and standard deviations. Stars indicate the significance of individual comparisons (* *p* < 0.05, ** *p* < 0.01, *** *p* < 0.001).

## Figures and Tables

**Table 1:** The effect of dexamethasone as well as adrenergic and muscarinic receptor agonists on the biosynthesis of PLs.

**Treatment**	**(D9)-PC**	**(D4)-PE**	**(D4)-PE P**	**(D9)-SM**	**(D9)-LPC**
	(% labeled)	(% labeled)	(% labeled)	(% labeled)	(% labeled)
	(nmol/mg)	(nmol/mg)	(nmol/mg)	(pmol/mg)	(pmol/mg)
**Control**	**13.6 ± 4.9**	**25.8 ± 2.7**	**9.4 ± 1.3**	**1.0 ± 0.3**	**4.5 ± 1.6**
	**12.8 ± 4.7**	**8.2 ± 1.6**	**2.2 ± 0.2**	**245 ± 74**	**65 ± 23**
**Dex**	11.4 ± 3.5**	21.3 ± 2.3 **	8.1 ± 1.2 *	0.6 ± 0.2 **	4.3 ± 1.1
	10.7 ± 3.1	6.6 ± 2.4	2.1 ± 0.4	162 ± 56	80 ± 26
**Control**	**6.0 ± 1.0**	**14.3 ± 1.5**	**7.0 ± 0.4**	**0.5 ± 0.0**	**2.0 ± 0.1**
	**4.6 ± 0.8**	**2.9 ± 0.6**	**2.8 ± 0.3**	**115 ± 28**	**26 ± 3**
**Dex**	5.4 ± 1.8	11.3 ± 1.3 **	6.8 ± 0.6	0.3 ± 0.0 ***	2.1 ± 0.4
	4.0 ± 1.4	2.1 ± 0.3	2.7 ± 0.4	87 ± 22	27 ± 7
**Dex + RU 486**	5.9 ± 1.6	12.4 ± 1.5	7.3 ± 0.7*	0.4 ± 0.0 **	2.0 ± 0.4
	4.5 ± 1.4	2.3 ± 0.4	3.0 ± 0.4	105 ± 32	27 ± 8
**Control**	**11.8 ± 3.2**	**22.6 ± 2.9**	**8.0 ± 1.9**	**0.9 ± 0.2**	**3.9 ± 1.2**
	**11.9 ± 3.8**	**7.6 ± 2.6**	**2.1 ± 0.4**	**222 ± 61**	**59 ± 20**
**Terbutaline**	12.5 ± 4.1	23.8 ± 1.8	8.4 ± 1.1	0.8 ± 0.2	5.0 ± 1.3 *
	11.5 ± 4.7	7.1 ± 1.2	2.0 ± 0.3	202 ± 69	74 ± 23
**Epinephrine**	12.8 ± 4.9	24.2 ± 2.3	8.8 ± 1.3	0.9 ± 0.2	5.4 ± 1.6 *
	11.3 ± 4.3	7.2 ± 1.4	2.0 ± 0.1	197 ± 63	75 ± 26
**Carbachol**	12.5 ± 4.8	24.1 ± 2.8	8.5 ± 1.9	0.9 ± 0.3	4.7 ± 1.7
	12.1 ± 5.7	7.7 ± 2.8	2.1 ± 0.6	217 ± 92	81 ± 38
**Pilocarpine**	12.8 ± 5.4	24.5 ± 2.7 *	8.6 ± 1.7	0.9 ± 0.3	5.3 ± 2.0**
	12.5 ± 4.9	8.4 ± 3.7	2.2 ± 0.7	237 ± 88	90 ± 36

Values obtained by ESI MS/MS for stable isotope-labeled PL classes were normalized to the cellular protein content and are expressed as nmol/mg protein or pmol/mg protein. For each PL class, the percentage of stable isotope-labeled PL from total labeled and unlabeled PL was calculated. Data are presented as means ± SD (*n* = 5–6). * *p* ≤ 0.05, ** *p* ≤ 0.01, *** *p* ≤ 0.001. Dex = dexamethasone; PC = phosphatidylcholine; PE = phosphatidylethanolamine; PE P = phosphatidylethanolamine-based plasmalogen; SM = sphingomyelin; LPC = lysophosphatidylcholine.

**Table 2:** The effects of dexamethasone as well as adrenergic and muscarinic receptors agonists on the release of PLs.

Treatment	[^3^H]-Choline-Labeled PLs (% Released PLs)	[^14^C]-Ethanolamine-Labeled PLs (% Released PLs)
**Control**	**4.97 ± 1.04**	**2.19 ± 0.65**
**Dexamethasone**	4.80 ± 1.44	2.09 ± 0.69
**Control**	**5.03 ± 0.78**	**1.79 ± 0.33**
**Terbutaline**	4.97 ± 1.20	2.06 ± 0.57
**Epinephrine**	5.08 ± 1.18	ND
**Carbachol**	4.67 ± 1.22	2.14 ± 0.68
**Pilocarpine**	5.19 ± 1.55	ND

Using our novel in vitro model, the release of radiolabeled lipids from confluent FLS into media over a 24 h period was monitored in the presence of 10 µM dexamethasone, terbutaline, epinephrine, carbachol, or pilocarpine. The quantitative dpm-values were normalized to the cellular protein content. The data presented are the percentages of radiolabeled PLs being released from total radiolabeled PLs as found in media and cellular lysates which were calculated separately for the [^3^H]- as well as for the [^14^C]-labeled PLs. The data are expressed as means ± SDs (*n* = 4–6). ND = not determined.

## Supplementary Materials

Supplementary materials can be found at http://www.mdpi.com/1422-0067/20/2/342/s1.

## Abbreviations

COX	Cyclooxygenase
DPPC	Dipalmitoyl-Phosphatidylcholine
FLS	Fibroblast-like Synoviocytes
IL-1ß	Interleukin-1ß
LPC	Lysphosphatidylcholine
MS	Mass Spectrometry
OA	Osteoarthritis
PC	Phosphatidylcholine
PE	Phosphatidylethanolamine
PE P	Phosphatidylethanolamine-based plasmalogen
PL	Phospholipid
RA	Rheumatoid Arthritis
SM	Sphingomyelin
TNFα	Tumor necrosis factor α
