# Supplementary material for: The Effect of Dexamethasone, Adrenergic and Cholinergic Receptor Agonists on Phospholipid Metabolism in Human Osteoarthritic Synoviocytes
Source: Int J Mol Sci. 2019 Jan 15;20(2):342. doi: 10.3390/ijms20020342 (PMC6359197; doi:10.3390/ijms20020342)
Supplement: Supplementary file 1 [file ijms-20-00342-s001.pdf]

Supplementary Material

The Effect of Dexamethasone, Adrenergic and Cholinergic Receptor Agonists on Phospholipid Metabolism in Human Osteoarthritic Synoviocytes

Katarzyna D. Sluzalska[1], Gerhard Liebisch[2], Bernd Ishaque[1], Gerd Schmitz[2], [1]Markus Rickert[1], and Juergen Steinmeyer[1]

[1]Laboratory for Experimental Orthopaedics, Department of Orthopaedics, Justus Liebig University Giessen, Paul-Meimberg-Str. 3, 35392 Giessen, Germany; K.Sluzalska@gmail.com; Bernd.Ishaque@ortho.med.uni-giessen.de; Markus.Rickert@ortho.med.uni-giessen.de

[2]Department of Clinical Chemistry and Laboratory Medicine, University Hospital Regensburg, Franz-Josef-Strauss-Allee 11, 93053 Regensburg, Germany; Gerhard.Liebisch@klinik.uni-regensburg.de; Gerd.Schmitz@klinik.uni-regensburg.de

Table S1. Effect of dexamethasone alone or in the presence of the glucocorticoid receptor antagonist RU 486 on the level of newly synthesized PL species.

PL species	Control		Dex		Dex + Ru 486	
	[pmol/mg]	[% labeled PL]	[pmol/mg]	[% labeled PL]	[pmol/mg]	[% labeled PL]
PC 30:0	48±10	4.8±0.7	35±12	**3.7±1.0**	42±12	4.4±0.9
PC 32:0	438±102	6.4±0.9	344±135	5.6±1.7	408±135	6.3±1.6
PC 34:0	59±11	6.3±1.1	43±20	5.1±1.8	56±27	6.0±1.9
PC 32:1	158±10	5.7±0.8	119±39	**4.6±1.5**	142±37	5.2±1.4
PC 34:1	861±90	6.0±1.4	726±206	5.3±2.1	831±185	5.8±1.9
PC 36:1	218±23	3.8±0.8	189±25	3.3±1.2	226±25	3.8±1.0
PC 34:2	358±34	9.0±1.7	275±88	**7.6±2.6**	332±79	8.8±2.3
PC 36:2	332±31	5.8±1.4	278±65	5.3±1.9	315±65	5.9±1.9
PC 34:3	39±5	10.9±1.2	28±8	9.4±3.0	33±8	10.1±1.9
PC 36:3	218±43	7.8±1.8	173±69	6.9±2.7	205±71	7.9±2.6
PC 38:3	84±24	4.9±0.7	82±31	5.0±1.5	85±34	5.1±1.4
PC 36:4	394±133	6.9±0.7	366±168	**6.1±1.3**	406±174	6.8±1.3
PC 38:4	482±186	5.3±0.7	474±246	5.1±1.5	515±248	5.6±1.2
PC 40:4	40±14	7.7±1.7	37±17	7.0±2.4	44±20	7.9±2.3
PC 36:5	52±17	8.0±1.6	46±19	7.5±1.4	51±21	7.4±1.7
PC 38:5	284±94	7.2±1.0	267±137	6.8±2.2	285±139	7.1±1.9
PC 40:5	60±19	7.0±1.6	58±29	6.7±2.6	63±28	7.0±2.2
PC 38:6	69±21	6.5±1.3	55±26	**5.6±1.8**	69±9	6.2±1.8
PC 40:6	42±15	6.1±1.3	39±20	5.7±1.9	46±21	6.3±2.0
SM 32:1	5±1	1.2±0.1	5±1	1.4±0.2	5±1	1.2±0.2
SM 33:1	5±1	0.9±0.2	4±1	0.6±0.1	4±1	0.7±0.2
SM 34:0	3±2	0.3±0.2	2±2	0.2±0.2	3±2	0.4±0.1
SM 34:1	54±8	0.4±0.0	37±11	**0.3±0.0**	46±8	**0.3±0.0**
SM 34:2	4±0	1.3±0.2	3±1	1.2±0.3	4±0	1.2±0.1
SM 35:2	7±1	1.0±0.3	9±2	1.3±0.7	7±1	0.9±0.2
SM 36:1	5±2	0.4±0.2	5±1	0.4±0.1	4±2	0.3±0.1
SM 36:2	3±1	2.1±0.6	2±0	1.5±0.1	2±1	1.6±0.2
SM 42:1	8±2	0.3±0.0	7±1	0.3±0.1	7±2	0.3±0.1
SM 42:2	12±4	0.5±0.1	6±2	**0.2±0.1**	12±5	**0.4±0.1**
PE 34:1	129±54	13.3±1.4	70±22	**8.9±1.8**	78±29	10.2±1.3
PE 36:1	122±67	8.8±1.3	83±31	**6.8±1.5**	99±46	8.0±1.2
PE 34:2	37±16	17.4±1.4	18±8	**12.2±4.2**	21±8	13.6±1.7
PE 36:2	108±42	13.3±1.9	66±19	**9.9±1.8**	73±28	10.8±2.2
PE 36:3	46±13	20.8±2.8	26±8	**15.5±3.5**	28±10	16.8±3.1
PE 38:3	208±42	16.1±1.9	155±19	**13.0±1.7**	160±26	14.4±1.7
PE 36:4	42±9	14.4±1.6	26±7	**10.7±2.0**	29±9	12.1±2.7
PE 38:4	722±134	9.4±1.4	558±125	**7.7±1.1**	574±142	8.0±1.8
PE 40:4	303±56	19.2±2.2	258±16	**17.0±2.1**	290±35	18.8±2.6
PE 38:5	288±67	14.7±2.6	183±54	**10.8±2.3**	194±52	12.1±2.5

PE 40:5	324±67	20.7±1.5	249±33	**17.5±1.8**	282±69	**19.3±1.9**
PE 38:6	198±44	26.6±3.7	106±30	**19.0±2.8**	130±42	22.4±4.4
PE 40:6	376±85	23.9±2.7	263±52	**18.9±2.3**	297±66	20.7±1.5
PE-P 16:0/18:1	48±17	3.7±0.3	42±13	3.1±0.6	55±21	**4.2±0.2**
PE-P 16:0/20:4	385±75	4.7±0.4	397±117	4.7±0.8	454±121	**5.4±1.0**
PE-P 16:0/22:4	158±29	9.5±1.6	159±10	9.4±0.9	158±20	9.0±1.0
PE-P 16:0/22:5	155±49	10.0±1.2	144±36	10.0±2.0	147±41	9.7±2.0
PE-P 16:0/22:6	119±16	8.3±1.5	100±27	7.4±0.7	119±27	8.6±1.4
PE-P 18:1/16:0	70±10	9.2±1.4	60±4	8.3±1.5	61±12	8.3±2.5
PE-P 18:1/18:1	54±6	9.2±2.2	53±15	8.4±1.0	60±17	9.6±2.0
PE-P 18:1/20:4	587±92	12.2±1.1	594±115	11.8±1.1	653±88	**13.0±0.8**
PE-P 18:1/20:5	62±7	17.8±3.3	61±6	17.1±3.0	64±12	16.3±0.9
PE-P 18:1/22:4	93±8	17.9±1.3	90±6	16.0±2.1	88±9	15.9±1.6
PE-P 18:1/22:5	80±16	17.3±1.8	70±8	14.8±2.0	89±22	**18.0±0.8**
PE-P 18:1/22:6	88±19	12.5±0.9	87±11	13.7±1.0	89±12	13.1±0.9
PE-P 18:0/16:0	43±5	15.2±4.8	43±3	15.0±3.3	38±6	13.6±3.6
PE-P 18:0/18:1	37±8	5.0±1.0	37±9	5.0±0.5	36±7	4.8±1.2
PE-P 18:0/20:4	461±113	4.2±0.4	456±116	4.2±0.7	550±149	**4.6±0.7**
PE-P 18:0/20:5	54±11	7.0±0.7	49±5	6.7±1.1	57±8	6.9±0.5
PE-P 18:0/22:4	88±18	7.5±1.8	82±21	7.0±1.0	92±10	7.0±1.4
PE-P 18:0/22:5	95±17	7.3±0.8	84±14	6.9±1.3	98±13	7.3±0.8
PE-P 18:0/22:6	85±15	5.7±0.6	73±14	5.5±0.6	90±8	6.1±1.1
LPC 16:0	10±1	1.7±0.2	11±4	1.7±0.4	11±3	1.7±0.3
LPC 18:0	7±2	3.4±0.6	7±2	3.2±1.0	8±3	3.4±1.2
LPC 18:1	8±1	1.8±0.2	9±2	2.1±0.2	9±2	1.8±0.2

The quantitative values obtained for each stable isotope-labeled PL species was normalized to the cellular protein content and expressed as pmol/mg cellular protein. For each PL species the percentage of stable isotope-labeled PL from total labeled and unlabeled PL was calculated. Data are presented as means ± SDs (n=5). Bold data correspond to significantly altered changes (expressed as fold) as shown in Figure 2 and 3. PC = phosphatidylcholine; PE = phosphatidylethanolamine; SM = sphingomyelin; PE-P = PE-based plasmalogen; LPC = lysophosphatidylcholine.

Table S2. Effect of adrenergic and muscarinic receptor agonists on the level of newly synthesized PL species.

PL species	Control		Terbutaline		Epinephrine	
	[pmol/mg]	[% labeled PL]	[pmol/mg]	[% labeled PL]	[pmol/mg]	[% labeled PL]
PE 34:1	373±215	20.9±4.3	328±113	21.5±2.7	344±123	21.7±3.4
PE 36:1	360±197	15.6±3.0	353±137	**17.5±2.7**	359±150	17.1±3.1
PE 34:2	102±53	25.4±3.5	90±27	25.8±2.6	97±31	26.4±2.8
PE 36:2	351±191	19.8±3.1	323±108	20.8±1.7	338±106	20.8±2.6
PE 36:3	128±62	28.0±4.5	111±29	28.1±2.9	114±34	27.0±3.1
PE 38:3	533±223	24.5±4.4	503±154	**26.1±3.7**	523±154	**26.6±4.3**
PE 36:4	100±45	22.6±4.1	90±23	22.3±1.9	100±27	25.2±3.9
PE 38:4	1786±694	16.2±2.6	1733±362	**17.7±1.9**	1713±411	17.8±1.8
PE 40:4	771±262	26.2±4.1	708±136	27.4±3.2	709±136	28.1±3.2
PE 38:5	722±322	20.5±3.2	676±154	21.6±1.5	708±171	22.6±2.3
PE 40:5	1024±216	31.2±4.1	977±94	33.0±2.9	946±110	33.0±4.4
PE 38:6	409±117	38.9±6.4	375±40	40.4±3.1	378±41	41.9±5.4
PE 40:6	920±147	35.8±4.9	853±103	36.9±3.4	832±103	38.0±4.2
LPC 16:0	17±4	2.8±0.8	20±6	3.4±1.0	23±9	4.3±1.3
LPC 18:0	21±9	4.1±1.6	30±10	**5.8±1.8**	29±11	**5.9±2.3**
LPC 18:1	21±8	5.8±1.5	24±8	6.6±1.3	24±10	6.6±1.6
**PL species**	**Control**		**Carbachol**		**Pilocarpine**	
	[pmol/mg]	[% labeled PL]	[pmol/mg]	[% labeled PL]	[pmol/mg]	[% labeled PL]
PE 34:1	373±215	20.9±4.3	375±215	21.6±3.9	419±258	22.4±3.3
PE 36:1	360±197	15.6±3.0	402±256	**17.5±3.7**	425±248	17.4±3.3
PE 34:2	102±53	25.4±3.5	108±61	27.0±3.6	117±70	26.9±4.2
PE 36:2	351±191	19.8±3.1	373±225	21.0±3.3	416±246	21.4±3.0
PE 36:3	128±62	28.0±4.5	123±61	27.7±3.2	141±77	28.8±3.6
PE 38:3	533±223	24.5±4.4	565±242	**27.7±4.1**	592±263	**27.0±3.9**
PE 36:4	100±45	22.6±4.1	103±53	23.5±4.6	110±52	23.3±2.6
PE 38:4	1786±694	16.2±2.6	1849±739	17.6±2.6	2100±946	**18.5±2.7**
PE 40:4	771±262	26.2±4.1	760±297	27.6±4.1	828±303	**28.8±4.1**
PE 38:5	722±322	20.5±3.2	746±352	21.9±3.1	835±417	22.6±3.4
PE 40:5	1024±216	31.2±4.1	1003±179	32.9±4.0	1063±415	33.3±5.1
PE 38:6	409±117	38.9±6.4	403±98	41.5±5.3	413±167	40.5±4.7
PE 40:6	920±147	35.8±4.9	910±126	38.3±4.6	955±343	38.6±4.0
LPC 16:0	17±4	2.8±0.8	24±11	3.4±1.3	27±9	4.0±1.1
LPC 18:0	21±9	4.1±1.6	31±16	5.2±2.2	35±16	**5.8±2.8**
LPC 18:1	21±8	5.8±1.5	27±12	5.9±1.8	28±12	6.7±2.4

The quantitative values obtained for each stable isotope-labeled PL species was normalized to the cellular protein content and expressed as pmol/mg cellular protein. For each PL species, the percentage of stable isotope-labeled PL from total labeled and unlabeled PL was calculated. Data are presented as means ± SDs (n=5-6). Bold data correspond to significantly altered changes (expressed as fold, $p \leq 0.05$) as described in the results section. PE = phosphatidylethanolamine; LPC = lysophosphatidylcholine.
